# Treatment with Autophagy Inducer Trehalose Alleviates Memory and Behavioral Impairments and Neuroinflammatory Brain Processes in db/db Mice

**DOI:** 10.3390/cells10102557

**Published:** 2021-09-27

**Authors:** Tatiana A. Korolenko, Nina I. Dubrovina, Marina V. Ovsyukova, Nataliya P. Bgatova, Michael V. Tenditnik, Alexander B. Pupyshev, Anna A. Akopyan, Natalya V. Goncharova, Chih-Li Lin, Evgeny L. Zavjalov, Maria A. Tikhonova, Tamara G. Amstislavskaya

**Affiliations:** 1 Scientific Research Institute of Neurosciences and Medicine, Timakova St. 4, 630117 Novosibirsk, Russia; dubrov@physiol.ru (N.I.D.); maryov@physiol.ru (M.V.O.); m.v.tenditnik@physiol.ru (M.V.T.); apupyshev@mail.ru (A.B.P.); akopyanaa@physiol.ru (A.A.A.); natalya15.ru@rambler.ru (N.V.G.); tikhonovama@physiol.ru (M.A.T.); amstislavskayatg@physiol.ru (T.G.A.); 2 Scientific Research Institute of Clinical and Experimental Lymphology—branch of the Federal Research Center “Institute of Cytology and Genetics”, Timakova St. 2, 630117 Novosibirsk, Russia; nataliya.bgatova@yandex.ru; 3 Institute of Medicine, Chung Shan Medical University, Taichung 40201, Taiwan; dll@csmu.edu.tw; 4 Federal Research Center “Institute of Cytology and Genetics”, Siberian Branch of the Russian Academy of Sciences, 630090 Novosibirsk, Russia; zavjalov@bionet.nsc.ru

**Keywords:** leptin-deficient db/db mice, trehalose, autophagy, behavior, memory, obesity, neurodegeneration

## Abstract

Autophagy attenuation has been found in neurodegenerative diseases, aging, diabetes mellitus, and atherosclerosis. In experimental models of neurodegenerative diseases, the correction of autophagy in the brain reverses neuronal and behavioral deficits and hence seems to be a promising therapy for neuropathologies. Our aim was to study the effect of an autophagy inducer, trehalose, on brain autophagy and behavior in a genetic model of diabetes with signs of neuronal damage (db/db mice). A 2% trehalose solution was administered as drinking water during 24 days of the experiment. Expressions of markers of autophagy (LC3-II), neuroinflammation (IBA1), redox state (NOS), and neuronal density (NeuN) in the brain were assessed by immunohistochemical analysis. For behavioral phenotyping, the open field, elevated plus-maze, tail suspension, pre-pulse inhibition, and passive avoidance tests were used. Trehalose caused a slight reduction in increased blood glucose concentration, considerable autophagy activation, and a decrease in the neuroinflammatory response in the brain along with improvements of exploration, locomotor activity, anxiety, depressive-like behavior, and fear learning and memory in db/db mice. Trehalose exerted some beneficial peripheral and systemic effects and partially reversed behavioral alterations in db/db mice. Thus, trehalose as an inducer of mTOR-independent autophagy is effective at alleviating neuronal and behavioral disturbances accompanying experimental diabetes.

## 1. Introduction

Diabetes mellitus is a prevalent metabolic disorder contributing to significant morbidity and mortality in humans [1,2]. Diabetes development is closely related to progressive hyperglycemia, leading to the formation of advanced glycation end products. The latter, when interacting with a receptor called RAGE, triggers a release of proinflammatory cytokines and inflammation that spread to many tissues and organs [3,4]. Many preventative and therapeutic agents have been developed for normalizing the glycemic profile in patients with diabetes. In addition to various pharmacological treatments, nonpharmacological modalities have been suggested to improve glycemic control in patients with diabetes [5,6]. In some experiments, amyloid-beta pathologies link Alzheimer’s disease and type 2 diabetes in a transgenic model [7,8]. A model of type 2 diabetes mellitus, called db/db mice, is characterized by a deletion of the leptin receptor gene; this deficit leads to obesity, insulin resistance, and type 2 diabetes mellitus [9].

A variety of diabetic complications have been observed in various tissues of the peripheral and central nervous systems. Cognitive dysfunction is a major complication in type 2 diabetes mellitus [7,8,9,10,11]. Diabetes causes such complications as insulin resistance, heart and kidney failure, neurodegeneration, and Alzheimer’s disease [2]. According to numerous studies, db/db mice are regarded as a classic model of diabetes-mellitus-mediated cognitive dysfunction. Several mechanisms are thought to be responsible for cognitive deficits in diabetes, including impaired hippocampal synaptic plasticity, decreased density of dendritic spines, oxidative stress, and inflammation. Dysregulated autophagy is involved in the pathogenesis of various neurodegenerative diseases such as Alzheimer’s disease and Parkinson’s disease. Nonetheless, the relation between autophagy and cognitive dysfunction in db/db mice remains unclear. Thus, in this study, we hypothesized that reduced autophagy might be involved in the behavioral dysfunction seen in db/db mice and that stimulation of autophagy could reduce the deficit.

Trehalose, a disaccharide of glucose, is a naturally occurring nontoxic and nonreducing bioactive sugar that is synthesized in many organisms when cells are exposed to stressful conditions, including dehydration, heat, oxidation, hypoxia, or even anoxia. Trehalose is not synthesized in the human body but is widely used in the food industry. Trehalose, which is synthesized in many organisms ranging from bacteria to plants, may modulate insulin sensitivity via more than seven molecular pathways, thus leading to better control of hyperglycemia [12,13]. Although the exact cause of insulin resistance has not been fully elucidated, a number of major mechanisms have been uncovered, namely, oxidative stress, inflammation, insulin receptor mutations, endoplasmic-reticulum stress, and mitochondrial dysfunction [12].

The relation between neurodegeneration and diabetes is determined by the similarity of the pathogenic factors that cause these processes [14,15]. Neurodegenerative diseases (Alzheimer’s disease, Parkinson’s disease, and Huntington’s disease) involve oxidative stress in their pathogenesis through a variety of mechanisms, including induction of oxidation of nucleic acids, proteins, and lipids; formation of advanced glycation end products; mitochondrial dysfunction; glial-cell activation; amyloid β deposition and plaque formation; apoptosis; cytokine production; inflammatory responses; and proteasome dysfunction [14,16]. Not all aspects of neurodegeneration development have been studied thoroughly to date, including the behavioral characteristics of experimental animals with diabetes [17,18,19]. The relation of the state of autophagy with damage to the hippocampus and the progression of cognitive impairment remains poorly understood. Due to the multiple mechanisms underlying the development of type 2 diabetes mellitus in db/db mice, which are burdened by the signs of Alzheimer’s disease and cognitive impairment, the disaccharide trehalose was chosen here as a suitable therapy and has shown its effectiveness in the treatment of neurodegeneration [20,21].

In this regard, the aim of the study was to investigate the effect of autophagy inducer trehalose on behavior, memory, and brain autophagy in db/db mice, which feature metabolic disorders related to leptin receptor deficiency followed by neuroinflammatory disorders and obesity and are burdened by cognitive dysfunction.

## 2. Materials and Methods

### 2.1. Animal Used

The study was carried out on specific pathogen-free mice of db/db strain (BKS.Cg- Dock7m+/+Leprdb/J, stock #000642; Jackson Laboratory; Bar Harbor, ME, USA). Wild-type (WT) C57BL/6J male mice and db/db mice (3 months old), which had leptin receptor deficiency, were purchased from the SPF-vivarium of the Institute of Cytology and Genetics SB RAS (Novosibirsk, Russia). The animals were housed on a regular 12/12 h light/dark cycle (lights on 02:00 a.m.), a constant room temperature of 23 ± 2 °C, and relative humidity of 45 ± 10%. The mice had access to standard mouse chow and water *ad libitum* during the 2-week acclimation period prior to experimentation. All experimental procedures were conducted according to the guidelines of the Declaration of Helsinki and the eighth edition of the Guide for the Care and Use of Laboratory Animals, published in 2011 by the United States National Academy of Sciences. The study protocol was approved by the Local Ethics Committee of the Scientific Research Institute of Neurosciences and Medicine (approval No. 2 of 20 February 2020) and the Institutional Review Board (7 July 2021).

### 2.2. Experimental Design

Mice were subdivided into four groups (6–8 animals each): (1) WT mice drinking water ad libitum during the whole experiment (24 days); (2) WT mice drinking 2% trehalose solution instead; (3) db/db mice drinking water ad libitum during the whole experiment; (4) db/db mice drinking 2% trehalose solution instead. Behavior testing began two weeks after the start of the experiment (on days 15–24) without stopping the consumption of trehalose. On the day of euthanasia (day 25), animals of all groups were culled with CO_2_ and transcardially perfused as described below (Section 2.5). The scheme of the experiment is given in Supplementary Figure S1.

### 2.3. Biochemical Assays

The blood samples were collected during chest dissection prior to transcardial perfusion. The blood collection, serum preparation and storage, and biochemical assays in the serum were performed as described earlier [22]. Total serum cholesterol and triglycerides (TGs) were quantified on an Architect c8000 biochemical analyzer (Abbott, Abbott Park, IL, USA), whereas low-density lipoprotein (LDL) cholesterol was assayed with the Biosystems kits, Costa Brava (Biosystems S.A., Barcelona, Spain) on a Labio 200 biochemical analyzer BS-200 (Shenzhen Mindray Bio-Medical Electronics Co., Ltd., Shenzhen, China) [23,24].

### 2.4. Behavioral Tests

#### Pre-Pulse Inhibition Test (PPI)

The PPI test of the acoustic startle reflex (ASR) was performed in a sound-attenuating chamber SR-Lab-San Diego Instruments, San Diego, CA, USA, Startle Response System. A session was initiated with an acclimation period followed by 10 presentations of startle pulses-alone (110 dB)—Blocks 1. Trial types for the PPI included four different pre-pulse intensities (72, 78, 82, and 86 dB); each pre-pulse preceded the startle pulse (110 dB) by a 40 ms inter-stimulus interval and was presented 10 times in random order (Blocks 2 and 3). Inter-trial intervals varied from 5 to 25 s. Block 4 contained the exact same 10 startle pulses trials as Block 1 [25].

We calculated PPI for each pre-pulse intensity as follows: PPI = 100 × [(pulse-alone) − (pre-pulse + pulse score)]/pulse-alone score, where the pulse-alone score is the average of pulse-alone values from Blocks 2 and 3.

#### The Open-Field Test (OFT)

The OFT was carried out in an apparatus with a square arena (40 cm × 40 cm) and plastic walls 37.5 cm high brightly lit from above (1000 lux). For 10 min, the animal was free to explore the test arena. The following parameters were determined: general locomotion (the distance traveled in cm); vertical locomotor and exploratory activity (rearing number); anxiety (time spent in the central part of the arena and the number of entries into the center) [26,27].

#### Elevated Plus Maze (EPM) Test

The EPM apparatus consisted of two open arms (30 × 5 cm), two closed arms of the same size with 15 cm-high walls, and a center platform (5 × 5 cm), and was elevated to a height of 120 cm above the test room floor. Each mouse was placed in the central area facing one of the open arms. Distance traveled, the number of entries, and the percentage of time time spent in the center or in open or closed arms were recorded for 5 min. The apparatuses were cleaned with 75% alcohol after each test. A reduction of the percent of time spent and the number of entries into the center and open arms is considered an anxiety-like index [18].

#### Tail Suspension Test (TST)

The TST was performed to evaluate depression-like and antidepressant behavior after drug treatment [28,29]. A mouse’s tail was threaded through the opening of the plastic panel and secured with a patch. The test was conducted for a 6 min period. Depressive-like behavior was inferred from increased duration of immobility. Immobility was defined as a lack of attempt to move limbs and staying in the vertical posture during the suspension.

Behavioral indicators in the OFT and TST were recorded using vertical and horizontal video cameras and the EthoVisionXT software and hardware system (Noldus, Wageningen, The Netherlands).

#### Passive Avoidance Test

Fear memory and learning were assessed via the passive avoidance paradigm as previously described [25,29]. A single conditioning session was performed in an experimental chamber consisting of dark and light compartments of the automated Gemini Avoidance System (San Diego Instruments, San Diego, CA, USA). A mouse received a foot shock (0.5 mA, 2 s) after entering the dark compartment, and the door was closed to prevent an escape on the training day. The number of crossings between the compartments at habituation and latencies to enter into the dark compartment on the training and test days were automatically recorded by the GEMINI. San Diego Instruments.Version 1.0.0 (San Diego Instruments, San Diego, CA, USA). The maximum duration of testing was 300 s.

### 2.5. Immunohistochemical (IHC) Analysis

The animals were transcardially perfused with phosphate-buffered saline (PBS) and 4% paraformaldehyde in PBS. The brains were postfixed in a 30% sucrose solution in PBS at 4 °C, then immersed in the embedding Tissue-Tek O.C.T. compound (Sakura Finetek USA, Torrance, CA, USA), frozen, and stored at −70 °C.

The IHC analysis was performed on 30 μm-thick cryosections according to a protocol described in detail previously [26,30,31]. Coronal slices along the hippocampus and amygdala (AP: −2.03 to −2.15 mm) of each mouse brain were prepared. We applied a rabbit polyclonal antibody (NB100-2220, 1:400 dilution, Novus Biologicals, Littleton, CO, USA) as a primary antibody to detect autophagosome marker MAP1LC3B, a mouse monoclonal antibody NOS-3F7-B11 B5 (GTX22801, 1:100 dilution, GeneTex, Alton Pkwy Irvine, CA, USA) as the primary antibody to detect nitric oxide synthase (NOS), a goat polyclonal antibody (NB100-1028, 1:200 dilution, Novus Biologicals, Littleton, CO, USA) as the primary antibody to detect the AIF-1/IBA1 microglial marker, and a rabbit monoclonal antibody (ab177487, 1:200 dilution, Abcam, Cambridge, UK) as the primary antibody to detect neuronal marker NeuN. A fluorescently labeled (Alexa Fluor 488—conjugated) goat anti-rabbit IgG antibody (ab150077, 1:500 dilution, Abcam, Cambridge, UK) served as the secondary antibody for LC3-II, IBA1, and NeuN, whereas a fluorescently labeled (Alexa Fluor 568—conjugated) goat anti-mouse IgG antibody (ab175473, 1:400 dilution, Abcam, Cambridge, UK) served as the secondary antibody for NOS.

Fluorescence intensity of IBA1 or NOS immunostaining was measured as background-corrected optical density (vs. background staining of the nonimmunoreactive regions) in the images converted to grayscale. Fluorescence intensity of punctate LC3-II was measured with subtraction of low diffuse fluorescence of some areas (punctate staining vs. background staining of nonpunctate regions) in the images converted to grayscale.

The density of neurons was determined in the frontal cortex and hippocampal regions CA1 and CA3 as the area occupied by NeuN-positive cells.

Fluorescent images were finally obtained by means of an Axioplan 2 (Carl Zeiss, Jena, Germany) imaging microscope and then analyzed in Image Pro Plus Software 6.0 (Media Cybernetics, Rockville, MD, USA). The area of interest was 19,353 μm^2^ in the hippocampal CA1 area, 26,100 μm^2^ in the hippocampal CA3 areas, and 18,208 μm^2^ in the dentate gyrus (DG), frontal cortex, and amygdala.

### 2.6. Transmission Electron Microscopy

Liver samples for electron microscopy were fixed in 4% paraformaldehyde in Hanks medium and a 1% OsO_4_ solution (Sigma, St. Louis, MO, USA) in phosphate buffer (pH 7.4) for 1 h, dehydrated in ethanol of ascending concentrations, and embedded in epon (Serva). Preparation of semithin sections and analysis under JEM 1400 electron microscope (JEOL Ltd.,Tokyo, Japan) have been described earlier [32].

### 2.7. Statistical Analysis

Results are presented as mean ± S.E.M. and were subjected to one-way, factorial, or repeated-measures ANOVA followed by post hoc Fischer’s LSD test. Data with *p* < 0.05 were regarded as statistically significant. The normality of the data distribution was determined by the Shapiro–Wilk *W* test.

## 3. Results

### 3.1. Effect of Trehalose Treatment on db/db Mice

**Figure 1:**
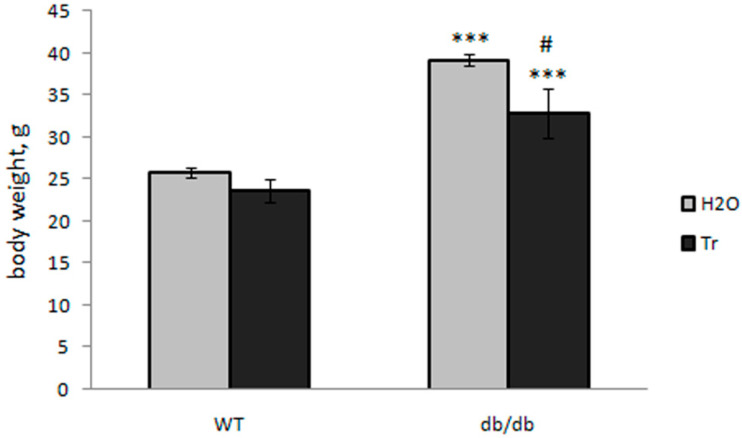
Effect of trehalose consumption with drinking water on body weight of db/db mice. The data are expressed as mean ± S.E.M of the values obtained in each group of animals (*n* = 6–8 per group). Statistically significant differences: *** *p* < 0.001 as compared to the control, ^#^ *p* < 0.05 as compared to the db/db-H_2_O group.

Compared to the control WT mice, the weight of db/db mice (drinking water) with obesity was higher; trehalose consumption with drinking water was followed by a decrease in body weight in db/db mice and to a lesser degree in WT mice (Figure 1).

**Figure 2:**
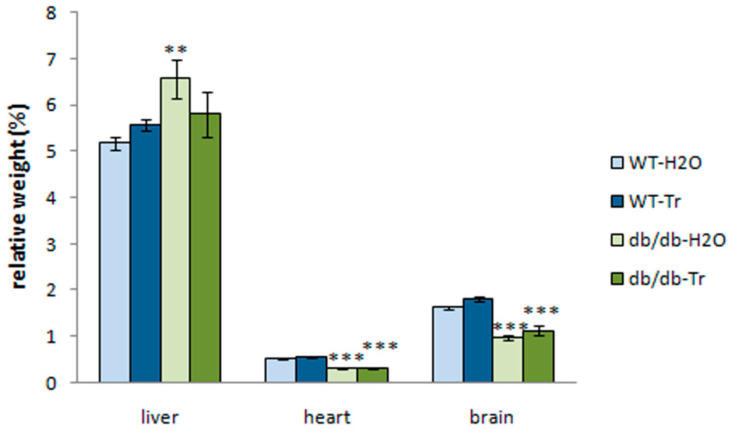
Relative weights of the liver, heart, and brain in db/db mice treated with trehalose. The data are expressed as the mean ± S.E.M. of the values obtained in each group of animals (*n* = 6–8 per group). Statistically significant differences: ** *p* < 0.01; *** *p* < 0.001 vs. WT.

Relative weight of the liver was higher in db/db mice (two-way ANOVA, genotype factor: F(1,25) = 5.71, *p* = 0.02); however, relative weights of the heart and brain were significantly lower (genotype factor: F(1,25) = 101.8, *p* < 0.0001; F(1,25) = 84.63, *p* < 0.0001, respectively). The drug factor (F(1,25) = 5.19, *p* = 0.03) produced a significant effect on the relative weight of their brains (Figure 2).

**Figure 3:**
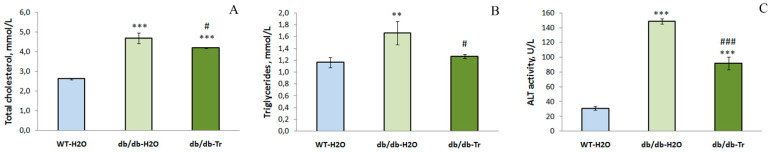
The impact of trehalose on serum total cholesterol (**A**), triglycerides (**B**), and ALT activity (**C**) in db/db mice. The data are expressed as the mean ± S.E.M. of the values obtained in each group of animals (*n* = 5–6 per group). Statistically significant differences: ** *p* < 0.01; *** *p* < 0.001 vs. WT-H_2_O group; ^#^ *p* < 0.05; ^###^ *p* < 0.001 vs. untreated db/db-H_2_O group.

Hypolipidemic effects of trehalose treatment were noted in db/db mice (Figure 3A,B), as was an improvement of liver function according to ALT activity (Figure 3C).

**Figure 4:**
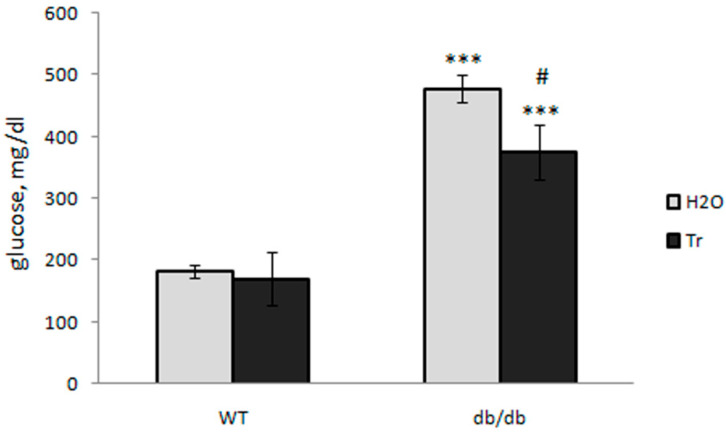
Effect of trehalose consumption with drinking water on the blood glucose level (mg/dL) in db/db mice. The data are expressed as the mean ± S.E.M. of the values obtained in each group of animals (*n* = 5–8 per group). Statistically significant differences: *** *p* < 0.001 vs. WT-H_2_O group, ^#^ *p* < 0.05 vs. db/db-H_2_O group.

Blood glucose was significantly higher in db/db mice than in control WT mice, and trehalose treatment decreased the blood glucose level compared to untreated db/db mice (Figure 4), but not compared to the normal level of WT mice.

### 3.2. IHC Analysis

**Figure 5:**
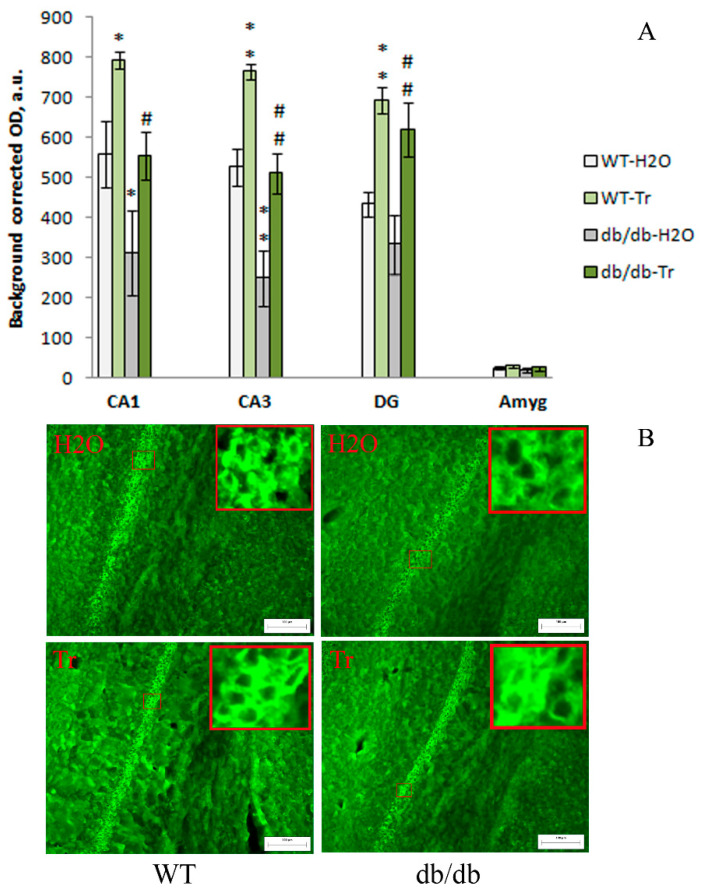
The impact of trehalose treatment on LC3-II expression in the hippocampus and amygdala. (**A**) Quantitative results. The data are expressed as the mean ± S.E.M. of the values obtained in each group of animals (*n* = 4 per group). Statistically significant differences: * *p* < 0.05; ** *p* < 0.01 vs. WT-H_2_O group; ^#^ *p* < 0.05; ^##^ *p* < 0.01 vs. untreated db/db-H_2_O group. (**B**) LC3-II immunoreactivity in the CA1 hippocampal area. Magnification, 200×; scale bar, 100 μm.

The genotype factor had a significant effect on the expression of LC3-II in the CA1 (F(1,12) = 10.6, *p* = 0.007) and CA3 (F(1,12) = 28.58, *p* = 0.0002) hippocampal areas. LC3-II expression in the CA1 (*p* < 0.05) and CA3 (*p* < 0.01) areas was lower in db/db mice than in the control WT mice (Figure 5).

The expression of LC3-II in the CA1 (F(1,12) = 10.6, *p* = 0.007), CA3 (F(1,12) = 25.29, *p* = 0.0003), and DG (F(1,12) = 25.05, *p* = 0.0003) hippocampal areas was significantly influenced by the drug factor. This parameter was markedly higher in all studied areas of the hippocampus of WT and db/db mice that consumed trehalose. Thus, compared to the control WT mice, brain LC3-II expression was lower in the tested hippocampal CA1 and CA3 areas of db/db mice, while trehalose treatment increased this index.

**Figure 6:**
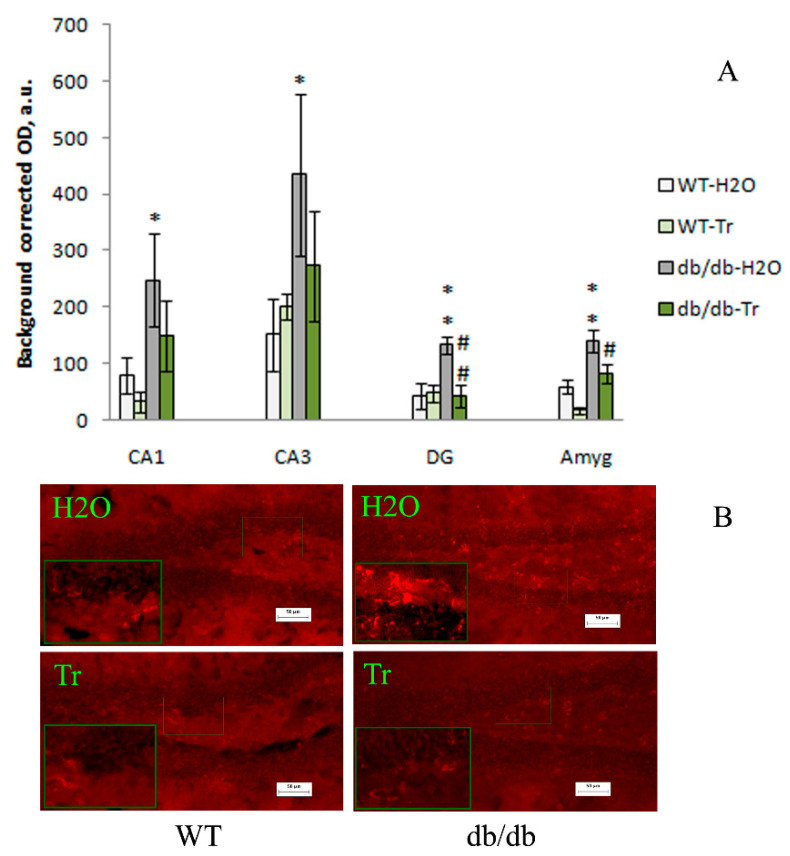
The influence of trehalose treatment on NOS expression in the hippocampus and amygdala. (**A**) Quantitative results. The data are expressed as the mean ± S.E.M. of the values obtained in each group of animals (*n* = 4 per group). Statistically significant differences: * *p* < 0.05; ** *p* < 0.01 vs. WT-H_2_O group; ^#^ *p* < 0.05; ^##^ *p* < 0.01 vs. db/db-H_2_O group. (**B**) NOS immunoreactivity in the DG of the hippocampus. Magnification, 200×; scale bar, 50 μm.

The expression of NOS was significantly influenced by the genotype factor in the CA1 (F(1,12) = 6.71, *p* = 0.02) and DG (F(1,12) = 5.09, *p* = 0.04) hippocampal areas and amygdala (F(1,10) = 21.8, *p* = 0.0009), and by the drug factor in the DG of the hippocampus (F(1,12) = 6.44, *p* = 0.03) and amygdala (F(1,10) = 9.96, *p* = 0.01). In db/db mice compared to the WT mice, NOS expression was higher in CA1 (*p* < 0.05), CA3 (*p* < 0.05), and DG (*p* < 0.01) areas of the hippocampus and amygdala (*p* < 0.01), while trehalose treatment decreased this index in the DG (*p* < 0.01) and amygdala (*p* < 0.05) (Figure 6). Thus, compared to the control WT mice, brain NOS expression was higher in all tested areas of the hippocampus (CA1, CA3, DG) and amygdala in db/db mice, while trehalose treatment significantly decreased this index in the DG and amygdala.

**Figure 7:**
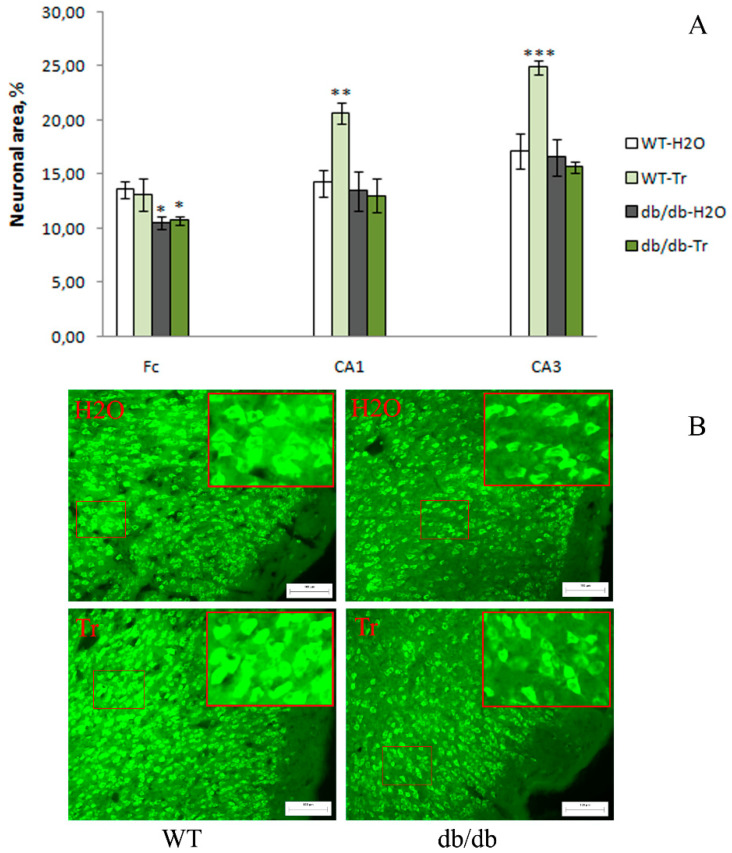
The influence of trehalose treatment on neuronal density (according to NeuN expression) in the frontal cortex (Fc) and CA1 and CA3 areas of the hippocampus. (**A**) Quantitative results. Neuronal density in the Fc of db/db mice was lower compared to the WT mice. The data are expressed as the mean ± S.E.M. of the values obtained in each group of animals (*n* = 4–5 per group). Statistically significant differences: * *p* < 0.05; ** *p* < 0.01; *** *p* < 0.001 vs. WT-H_2_O group. (**B**) Neuronal density according to NeuN expression in the Fc. Magnification, 200×; scale bar, 100 μm.

The neuronal nuclear antigen (NeuN) is a marker of neuronal maturation [30]. The density of neurons was determined in the prefrontal cortex and hippocampal regions CA1 and CA3 by means of the area occupied by NeuN-positive cells. Db/db mice were found to have a lower density of neurons in the frontal cortex (*p* < 0.05) but not in the hippocampus. Trehalose treatment did not affect neuronal density in the brain of db/db mice but increased this index in CA1 (*p* < 0.01) and CA3 (*p* < 0.001) areas of the hippocampus in WT mice (Figure 7). Thus, trehalose treatment did not influence the neuronal density in db/db mice but increased this index in the CA1 and CA3 areas of the hippocampus in WT mice.

**Figure 8:**
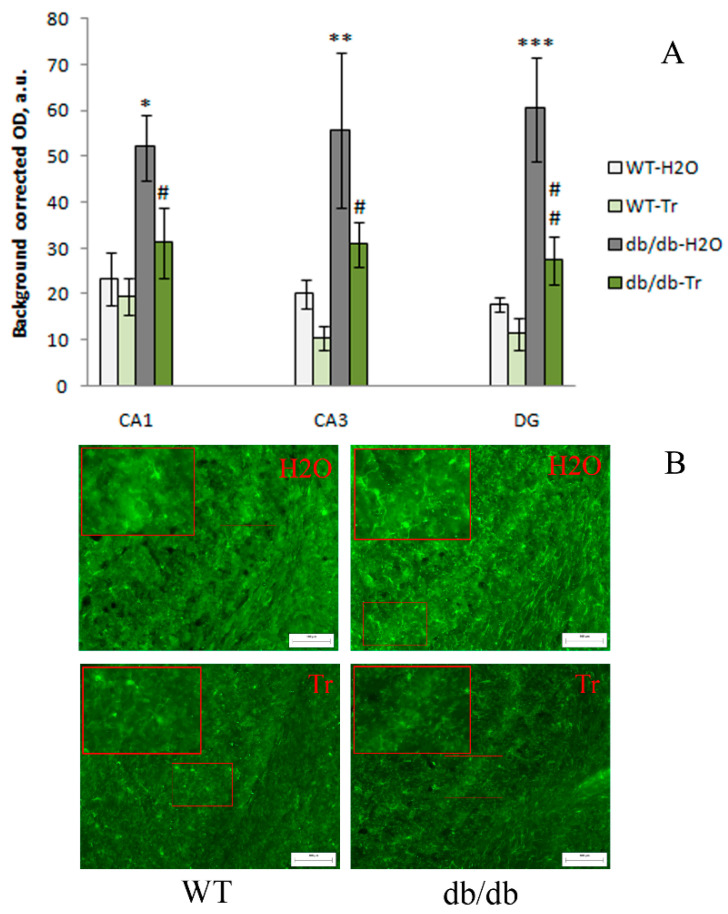
Effect of trehalose treatment on IBA1 expression in the hippocampus. (**A**) Quantitative results. The data are expressed as the mean ± S.E.M. of the values obtained in each group of animals (*n* = 4 per group). Statistically significant differences: * *p* < 0.05; ** *p* < 0.01; *** *p* < 0.001 vs. WT-H_2_O group; ^#^ *p* < 0.05; ^##^ *p* < 0.01 vs. db/db-H_2_O group. (**B**) IBA1 immunoreactivity in hippocampal CA1 area. Magnification, 200×; scale bar, 100 μm.

The expression of IBA1 was significantly influenced by the genotype factor in the CA1 (F(1,12) = 9.25, *p* = 0.01), CA3 (F(1,12) = 15,4, *p* = 0.002), and DG (F(1,12) = 26.58, *p* < 0.001) hippocampal areas, and by the drug factor in the CA3 area (F(1,12) = 5.82, *p* = 0.03) and DG of the hippocampus (F(1,12) = 12,01, *p* = 0.005). In db/db mice compared to WT mice, IBA1 expression was higher in the CA1 (*p* < 0.01), CA3 (*p* < 0.01), and DG (*p* < 0.001) areas of the hippocampus (*p* < 0.01), while trehalose treatment decreased this index (Figure 8). Thus, compared to the control WT mice, IBA1 expression was higher in hippocampal CA1 and CA3 areas and DG in db/db mice, while trehalose treatment decreased this index.

### 3.3. Behavioral Changes in db/db Mice and Protective Effects of Trehalose

#### 3.3.1. PPI of the ASR

**Figure 9:**
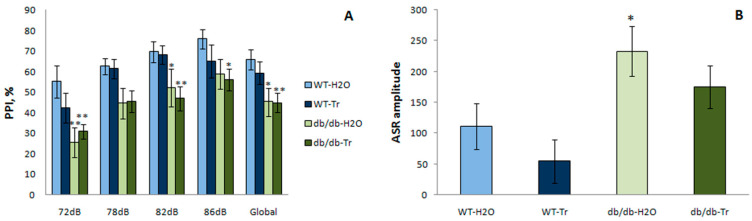
(**A**) Pre-pulse inhibition (PPI) and (**B**) the acoustic startle reflex (ASR) amplitude and the impact of trehalose (Tr). The data are expressed as the mean ± S.E.M. of the values obtained in each group of animals (*n* = 7–8 per group). Statistically significant differences according to the LSD post hoc test: ** *p* < 0.01; * *p* < 0.05 vs. WT-H_2_O group.

There was a significant genotype-affected difference between the db/db and WT mice in the PPI of the ASR (Figure 9). PPI was lower in db/db mice (two-way ANOVA, genotype factor) at 72 dB (F(1,25) = 9.72, *p* = 0.005), 78 dB (F(1,25) = 7.34, *p* = 0.01), 82 dB (F(1,25) = 13.98, *p* = 0.001), and 86 dB (F(1,25) = 4.05, *p* = 0.05), as well as globally across all prepulse intensities (F(1,25) = 9.86, *p* = 0.004). Trehalose did not have a significant influence on PPI in WT and db/db mice at all prepulse intensities (72, 78, 82, and 86 dB) and on the global prepulse (two-way ANOVA, drug factor: F(1,25) = 0.41, *p* = 0.53).

ASR was significantly higher in db/db than in WT mice (two-way ANOVA, genotype factor: F(1,25) = 10.7, *p* = 0.003). In mice that consumed trehalose, there was a tendency for a lower ASR (drug factor: F(1,25) = 2.4, *p* = 0.1).

#### 3.3.2. The OFT

**Figure 10:**
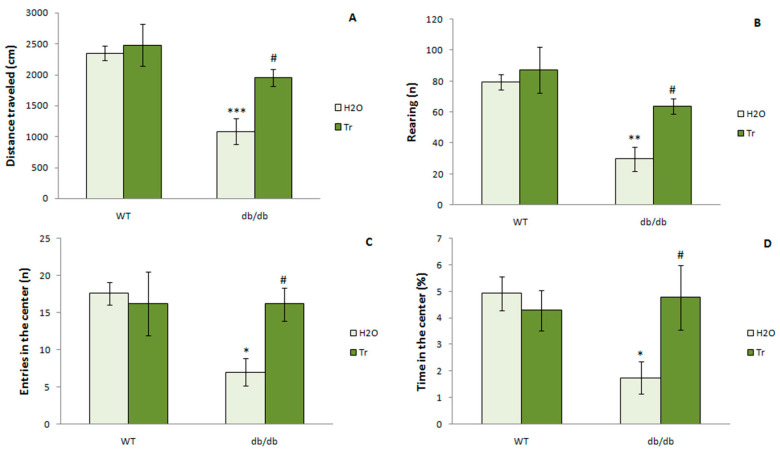
The effect of trehalose (Tr) on behavior of WT and db/db mice in the open field test. (**A**) Distance traveled; (**B**) rearings; (**C**) entries into the center; (**D**) time in the center. Each bar represents mean ± S.E.M. of data from 7–8 mice per group. Statistically significant differences according to the LSD post hoc test: * *p* < 0.05; ** *p* < 0.01; *** *p* < 0.001 vs. WT-H_2_O group, ^#^ *p* < 0.05 vs. db/db-H_2_O.

The OFT showed a reduction in overall exploratory/locomotor activity in the diabetic mice. The db/db mice traveled shorter total distances (*p* < 0.01) in the arena and had a lower rearing frequency (*p* < 0.01) than WT mice (Figure 10). After treatment with trehalose, db/db mice showed an improvement in exploratory/locomotor activity. They traveled a longer distance (*p* < 0.05) and made more rearings (*p* < 0.05) than db/db mice that consumed water.

Anxiety-like behaviors were noted in the OFT performed on diabetic mice. Compared with the WT, db/db mice spent less time in (*p* < 0.05) and entered the central area less often (*p* < 0.05). Trehalose attenuated anxiety-like behavior in the mice with diabetes; the db/db mice that consumed trehalose spent more time in the center (*p* < 0.05) and visited it more often (*p* < 0.05) than the db/db mice that drank water. We can conclude that trehalose treatment increases motor and exploratory activity and reduces anxiety in db/db mice.

#### 3.3.3. EPM Test

**Figure 11:**
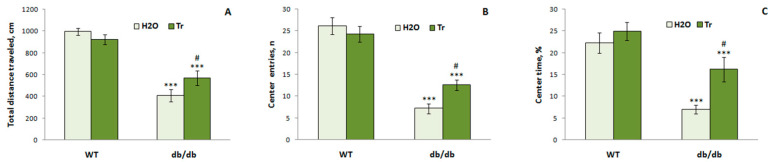
The impact of trehalose (Tr) on behavior of WT and db/db mice in the EPM test. Total distance traveled (**A**), the number of entries (**B**), and percentage of time spent (**C**) in the center of the elevated plus-maze. Each bar represents mean ± S.E.M. of data from 7–8 mice per group. Statistically significant differences according to the LSD post hoc test: *** *p* < 0.001 vs. WT-H_2_O group, ^#^ *p* < 0.05 vs. db/db-H_2_O group.

This test also showed a reduction in exploratory/locomotor activity and increase in anxiety in the diabetic mice (Figure 11). Db/db mice traveled shorter total distances (F(1,24) = 79.66, *p* < 0.0001), and there were less frequent passages between arms (F(1,24) = 62.24, *p* < 0.0001). They went out into the open arms (F(1,24) = 28.99, *p* < 0.0001) and the central area of the maze less often (F(1,24) = 474.45, *p* < 0.0001), and spent less time in the center (F(1,24) = 27.8, *p* < 0.0001) and more time in the closed arms (F(1,24) = 21.55, *p* < 0.0001).

Treatment with trehalose led to an increase in exploratory/locomotor activity and a decrease in the manifestation of anxiety-like behaviors in db/db mice. Db/db mice that consumed trehalose traveled a longer distance (genotype x drug factor: F(1,24) = 4.99, *p* < 0.05); they often went to the center of the maze (genotype x drug factor: F(1,24) = 1.14, *p* < 0.05) and spent more time there (drug factor: F(1,24) = 6.67, *p* < 0.05) but less time in the closed arms (drug factor: F(1,24) = 6.22, *p* < 0.05) than the db/db mice that consumed water. The influence of the genotype x drug factor on the number of passages between arms was detected (F(1,24) = 4.51, *p* < 0.05). Thus, we showed that trehalose reduced manifestations of anxious behavior.

#### 3.3.4. TST

**Figure 12:**
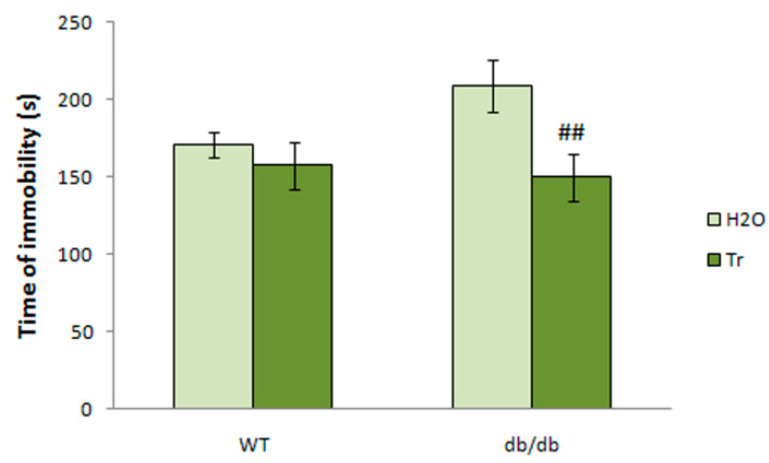
The effect of trehalose (Tr) on depressive-like behavior of WT and db/db mice in the tail suspension test. The data are expressed as the mean ± S.E.M. of the values obtained in each group of animals (*n* = 6–8 per group). Statistically significant differences according to the LSD post hoc test: ^##^ *p* < 0.01 vs. db/db-H_2_O group.

There was a tendency for longer immobility in the TST in db/db mice (*p* < 0.1 vs. WT). Treatment with trehalose reduced the depressive-like behavior in the db/db mice by reducing the duration of immobility (*p* < 0.01 vs. db/db-H_2_O group; Figure 12). Therefore, trehalose treatment reduced depressive-like behavior in db/db mice.

#### 3.3.5. Passive Avoidance Test

**Figure 13:**
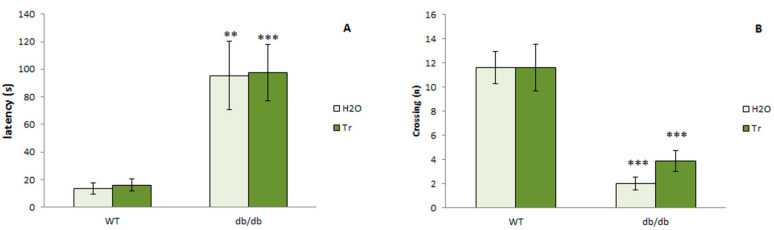
Hypolocomotive and hypoexploratory behavior of db/db mice during familiarization with an experimental chamber performed before a training session. (**A**) Latency to enter into the dark compartment. (**B**) crossing from one compartment into the next. Each bar represents mean ± S.E.M. of data from 7–8 mice per group. Statistically significant differences: ** *p* < 0.01; *** *p* < 0.001 vs. WT.

During the initial 300 s familiarization (habituation) period with an experimental chamber, deficient locomotor and exploratory activities in db/db mice were observed in comparison with WT mice (Figure 13). A two-way ANOVA detected the main effect of genotype (*p* < 0.001) but did not detect any significant effect of trehalose. Db/db mice expressed passive behavior as reflected by their longer latency to enter into the dark compartment (*p* < 0.01) and reduced number of crossings (*p* < 0.001) in comparison with WT mice. Trehalose did not change this behavior in either db/db or WT mice.

**Figure 14:**
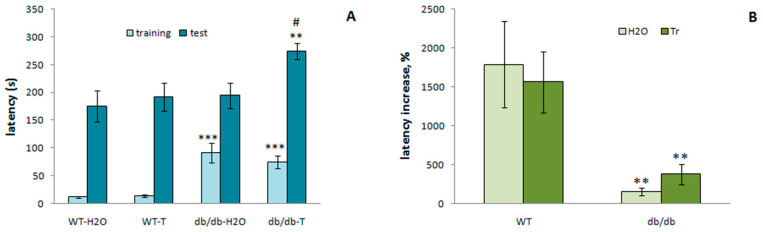
Trehalose attenuates impairment of the passive avoidance learning in db/db mice. (**A**) Latency to enter into the dark compartment. (**B**) crossing from one compartment into the next. Percentage of the increase in step-through latency on the test day relative to the training day. Each bar represents mean ± S.E.M. of data from 7–8 mice per group. Statistically significant differences according to the LSD post hoc test: ** *p* < 0.01; *** *p* < 0.001 vs. WT; ^#^ *p* < 0.05 vs. db/db-H_2_O group.

Figure 14 presents data on passive avoidance learning and the effects of trehalose in db/db and WT mice. It should be noted that db/db mice, on the day of training, retained a long latency to enter into the dark compartment compared to WT mice (*p* < 0.001), similarly to the day of familiarization. Retrieval of a memory trace was measured by means of step-through latency in the test session performed 24 h after training. In WT mice, long latency to enter into the fearful compartment was recorded during testing, indicating good retrieval of the fear memory trace. Db/db mice showed deficient learning in comparison with WT mice, as evidenced by the significance of the factor line in the ANOVA (*p* < 0.001) and by the significance of the percentage of increase in step-through latency on the test day relative to the training day (*p* < 0.001). Exposure to trehalose did not change the passive avoidance learning in WT mice; db/db mice treated with trehalose demonstrated pronounced avoidance of the fearful compartment during the testing period. A two-way ANOVA revealed that db/db mice that received trehalose had longer step-through latency than the db/db mice receiving H_2_O (*p* < 0.05). Therefore, trehalose corrected the deficit of the fear memory trace formation.

### 3.4. Morphological Analysis of Autophagy in the Liver

**Figure 15:**
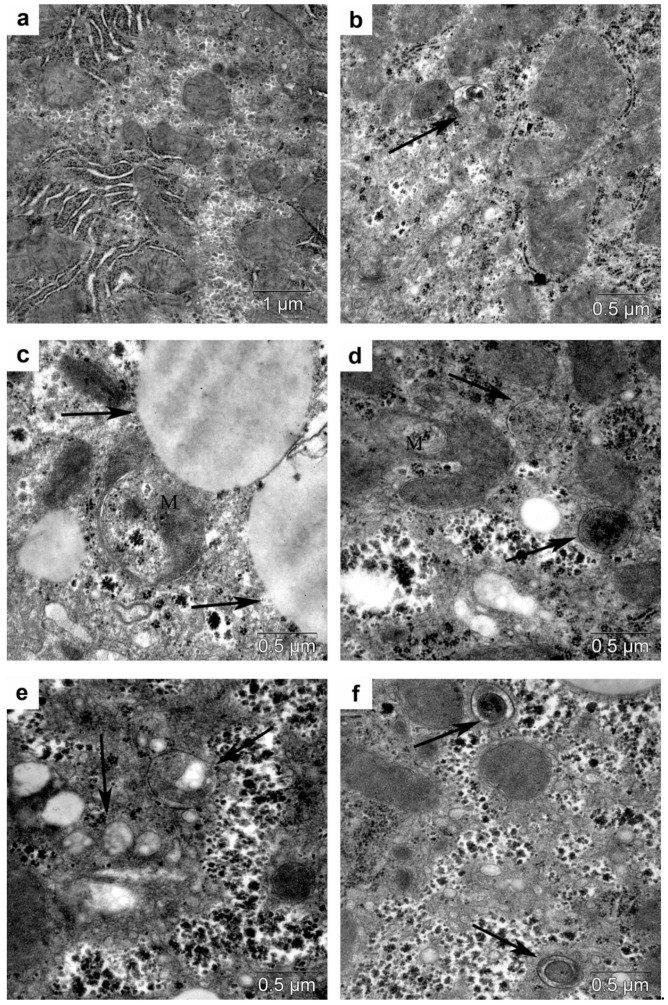
(**a**) A fragment of hepatocyte cytoplasm from a WT mouse. Mild glycogen accumulation and well-pronounced membranes of the endoplasmic reticulum and mitochondria. (**b**) A fragment of the cytoplasm of a hepatocyte from a WT mouse treated with trehalose. Autolysosome (arrow). (**c**) Accumulation of lipid droplets in the cytoplasm of a hepatocyte of a db/db mouse. Large lipid inclusions (arrows). Convoluted mitochondria (M). (**d**) An autophagosome with a cytoplasm fragment of a hepatocyte (arrows) from a db/db mouse treated with trehalose. Convoluted mitochondria (M). (**e**) An autophagosome with lipids in the cytoplasm of a hepatocyte from a db/db mouse treated with trehalose (arrows). (**f**) Autophagosomes with mitochondria in the cytoplasm of a hepatocyte from a db/db mouse treated with trehalose (arrows).

According to electron microscopic study of hepatocytes, results on intact WT mice are in agreement with the literature (Figure 15a). Trehalose consumption with drinking water was followed by the formation of autophagosomes in some hepatocytes (Figure 15b).

In db/db mice, significant accumulation of lipids was noted in the cytoplasm of hepatocytes. Hepatocyte heterogeneity in lipid content was also observed. The sizes of lipid inclusions were small, medium, and large (Figure 15c). Hepatocytes differed in the content of glycogen. Twisted and ring-shaped mitochondria were seen (Figure 15c,d). Autophagosomes with a cytoplasm fragment, mitochondria, and lipid droplets (LD) were noted (Figure 15d–f). Increased autophagy induced by trehalose was detectable in the brain and in liver cells.

## 4. Discussion

Neurodegenerative diseases are widespread disorders with a prevalence increasing with age. The effectiveness of their treatment depends on the understanding of their etiology, which is not clear at present. A common feature of these diseases is the accumulation of proteins prone to aggregation and protein inclusions, which are markers of these diseases and have pathogenic significance. The cellular tool for their elimination is autophagy (chaperone-mediated autophagy, macroautophagy, and selective autophagy). The key role of autophagy in the maintenance of cellular survival and in the suppression of neurodegeneration has been evaluated in Alzheimer’s, Parkinson’s, and Huntington’s diseases, which are accompanied by the accumulation of beta-amyloid, alpha-synuclein, and huntingtin, respectively. Autophagy is weakened in various ways in these diseases and decreases with aging [33]. Alzheimer’s disease and type 2 diabetes mellitus share many common pathophysiological features, such as insulin resistance, inflammation, oxidative stress, and amyloid aggregation and deposition [4].

Aberrant autophagy plays a key role in aging, type 2 diabetes mellitus, and neurodegeneration [34]. Suppression of autophagy in db/db mice partially resembles the changes that occur in aging [35]. Recent evidence indicates that polyphenols (e.g., resveratrol and curcumin), flavonoids (e.g., quercetin), polyamines (e.g., spermidine), and sugars (e.g., trehalose) limit brain damage in vitro and in vivo. Their common mechanism of action leads to the restoration of efficient autophagy, which subsequently processes the misfolded proteins and dysfunctional mitochondria [36]. Sodium-glucose cotransporter 2 (SGLT2) inhibitor empagliflozin and dipeptidyl peptidase 4 (DPP4) inhibitor linagliptin reactivate glomerular autophagy in db/db mice, thus manifesting a positive effect [37]. Recently, trehalose, an inducer of autophagy, was suggested as a possible positive factor in the control of neurodegeneration development and in the regulation of blood glucose levels [34,37]. Trehalose might also be used in the treatment of other diseases, such as cardiometabolic, liver, and other illnesses followed by disturbances in glucose metabolism [2,34,38]. Trehalose restores autophagy and some neuronal deficits induced by hyperglycemia [39,40,41]. Hence, trehalose as an inducer of mTOR-independent autophagy seems to be a promising agent for the treatment of neuronal and behavioral disturbances accompanying experimental diabetes. However, mechanisms of positive effects of autophagy inducers used in the treatment of diabetes in humans (metformin and trehalose) have been insufficiently studied.

Animal models and clinical assessments of diabetic patients have revealed that diabetes causes several abnormalities at both chemical and ultrastructural levels of the brain that result in dysfunctional behaviors [19] and increase the risk of major depressive disorder. In Alzheimer’s disease, there is predominant damage to the hippocampus, which is tightly linked with defects of cognition [42]. Although accumulating evidence indicates that oxidative stress, chronic inflammation, and programmed cell death may lead to the brain dysfunction associated with many neurological diseases, the precise mechanisms underlying diabetes-related neurological disorders remain unclear [43].

In the present study, we investigated the influence of trehalose on the cognitive abilities of db/db mice by analyzing behavioral, cognitive, and some biochemical characteristics of brain functions. The results revealed that trehalose attenuates the inflammatory response, increases autophagy, and improves cognitive function. It has been reported that db/db mice fail on some behavioral tasks, such as working/reference special memory in the Morris water maze test, novel object recognition task, and contextual and cued fear conditioning [11,44,45]. In the present study, diabetic mice showed impaired cognitive function as manifested in the passive avoidance test. We found a deficiency in the formation of a trace of fear memory. Db/db mice only poorly learned how to associate a conditioned stimulus (the environment context, an experimental chamber) with a mild foot shock (unconditioned stimulus), as evidenced by the significance of the percentage increase in latency to enter into the fearful compartment on the testing day relative to the training day.

Among the neuropsychiatric symptoms of diabetes, an important role belongs to emotional manifestations of behavior, such as anxiety, fear, attention, and depression, which make a significant contribution to the clinical profile of the disease. We demonstrated here that db/db mice are characterized by a decrease in motor and exploratory activity in terms of distance traveled and the number of rearings in the open field test. Db/db mice showed reduced motivation (a natural trait for rodents striving for a dark mink), as reflected by their long latency to enter into the dark compartment during habituation in the passive avoidance test and low exploratory activity, as reflected by the reduced number of crossings. In addition, these mice showed increased anxiety through shorter time and a lower number of visits into the central area in the open-field test and plus-maze test. They also tended to manifest depressive-like behavior as reflected by a tendency for longer immobility in the TST. An increase in the ASR was documented too, which corresponds to unconditioned fear, while a decrease in PPI for all pre-pulse intensities points to a deficit in sensorimotor gating, in selective attention, and in the filtering of insignificant information.

In db/db mice, we detected a functional disturbance in emotion that corresponds to Alzheimer’s-disease-like pathology [46], namely, anxiety in the elevated plus-maze and open field tests and its good alleviation due to trehalose application. There was also a recovery of exploratory and locomotor activity as assessed by the open field test. Db/db mice showed a deficit of long-term memory in the passive avoidance test, whereas trehalose improved this parameter. The trehalose treatment also had some restorative effect in the tail suspension test, which evaluates depressive-like behavior and antidepressant-like effects. Considerable alterations were seen in these mice in PPI and ASR testing in agreement with [18], but trehalose failed to reverse deficits of sensorimotor gating.

The inflammatory process and autophagy are crucial factors during the development of diabetes-associated cognitive impairment. We showed decreased expression of autophagy marker LC3-II in the hippocampus (CA1, CA3, and DG). Autophagy stimulation normalized the expression of LC3-II in db/db mice. In addition, the expression of endothelial nitric oxide synthase (NOS) and microglial activation (IBA1 expression) in the brain were greater in the diabetic mice and were attenuated by trehalose. It should be noted that the therapeutic effect of trehalose may be related to a beneficial action on inflammatory bowel processes, which are active in db/db mice and affect other concomitant complications, including brain disturbances [13,47]. It has been reported that activation of autophagy, in particular, by 3% trehalose, prevents inflammatory processes in the intestines of mice [48,49].

In db/db mice, defined by the deletion of the leptin receptor, there is uncontrolled food intake, obesity, hyperglycemia, insulin resistance, and the development of type 2 diabetes mellitus along with pathological complications, in particular, neurodegeneration and cognitive impairment. Here, the diabetic mice effectively reproduced the human symptoms (background obesity, decreased brain weight, and high blood glucose levels [50]), whereas long-term treatment with 2% trehalose caused a significant decrease in body weight (Figure 1) and blood glucose levels (Figure 4) without a significant influence on the weight of the brain (Figure 2).

Shortly after the application of trehalose, a short-term increase in the glucose level occurs in the blood, owing to the cleavage of trehalose by intestinal enzyme trehalase, yielding glucose [51]. The growth of glucose levels is prevented by simultaneous inhibition of glucose absorption in the intestine by trehalose [52]. In general, the normalizing effect on glucose metabolism is noted, consisting of an increase in insulin sensitivity and alleviation of metabolic syndrome [53,54].

Thus, in db/db mice, trehalose attenuated the negative manifestations of type 2 diabetes mellitus, judging by the detectable weight loss in the mice, a reduction in hyperglycemia, and a hypolipidemic effect. Moreover, trehalose exerted substantial beneficial actions on the exploratory and locomotor activity, anxiety, long-term memory, and depressive-like behavior. A good therapeutic effect of trehalose was documented here that is relevant to the pathogenesis of Alzheimer’s disease-like disturbances in the brain, in particular, inducible oxidative stress and neuroinflammation in the hippocampus, cognitive impairment, and behavioral alterations revealed in the passive avoidance test, OFT, EPM test, and tail suspension test, respectively. Due to these effects, trehalose can be considered a therapeutic agent for inhibiting neurodegenerative symptoms accompanying type 2 diabetes mellitus.

According to recent findings, alleviating effects of trehalose in diabetes can result from the improvement of glycemic control and insulin response [55], increased rate of autophagy [56,57], protective effect against oxidative stress [58], as well as influence on gut microbiota [59]. Therefore, trehalose was suggested as a potential preventive therapy for metabolic syndrome and diabetes development [60].

Here, we have shown a new positive effect of trehalose treatment on motor and exploratory activity, reducing anxiety, improving depressive-like behavior. The alleviation was associated with increased autophagy in the brain cells, a hypoglycemic effect, and improvement in immunohistochemical indexes (anti-inflammatory and anti-oxidative effects). As was revealed in our study, trehalose treatment induced autophagy not only in the brain cells but also in the liver (according to the increased number of autophagosomes in the hepatocytes) and possibly in other organs. Further studies are necessary and perspective in this area.

In summary, this study provides evidence that trehalose exerts protective effects against diabetes-induced cognitive and behavioral impairments by inhibiting the inflammatory response and enhancing autophagy. Thus, trehalose is expected to be a promising novel agent for the treatment of cognitive dysfunction in diabetes.

## 5. Conclusions

We demonstrated that trehalose has some beneficial peripheral and systemic effects and partially reverses behavioral aberrations in db/db mice. Trehalose, owing to a variety of cytoprotective mechanisms of action on a degenerating brain—with the main mechanism being autophagy induction—has a pronounced normalizing effect on the behavior and cognitive function of mice. Our results will provide the basis for in-depth research on the use of trehalose for the amelioration of diseases complicated by neurodegenerative processes.

## Data Availability

The data presented in this study are available on request from the corresponding author.

## Supplementary Materials

The following are available online at https://www.mdpi.com/article/10.3390/cells10102557/s1, Figure S1: Scheme of the experiment.
