# Supplementary material for: Treatment with Autophagy Inducer Trehalose Alleviates Memory and Behavioral Impairments and Neuroinflammatory Brain Processes in db/db Mice
Source: Cells. 2021 Sep 27;10(10):2557. doi: 10.3390/cells10102557 (PMC8533743; doi:10.3390/cells10102557)

cells

MDPI

Article

Treatment with Autophagy Inducer Trehalose Alleviates Memory and Behavioral Impairments and Neuroinflammatory Brain Processes in db/db Mice

Tatiana A. Korolenko [1,*], Nina I. Dubrovina [1], Marina V. Ovsyukova [1], Nataliya P. Bgatova [2], Michael V. Tenditnik [1], Alexander B. Pupyshev [1], Anna A. Akopyan [1], Natalya V. Goncharova [1], Chih-Li Lin [3], Evgeny L. Zavjalov [4], Maria A. Tikhonova [1] and Tamara G. Amstislavskaya [1]

Figure S1. Scheme of the experiment.

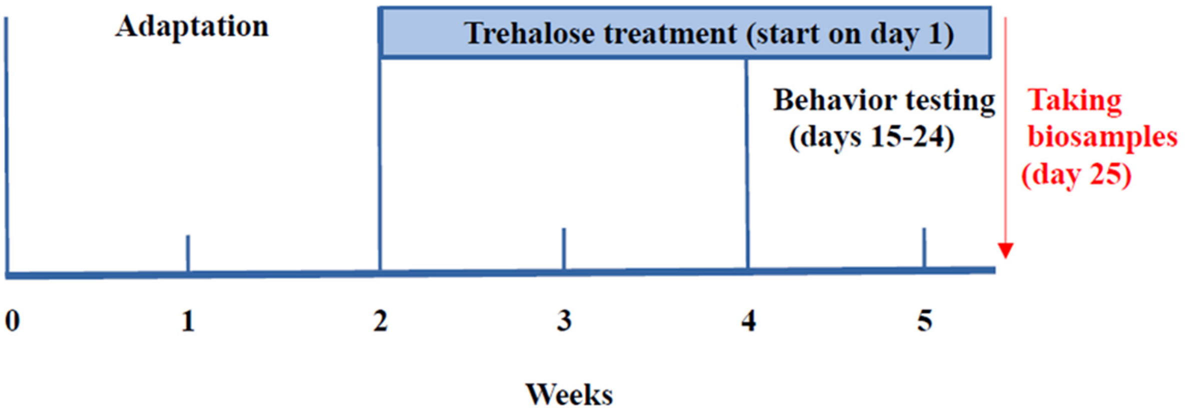

Supplement: Supplementary file 1 [file cells-10-02557-s001.zip › cells-1366361-supplementary.pdf]
